# An Okinawan-based Nordic diet improves anthropometry, metabolic control, and health-related quality of life in Scandinavian patients with type 2 diabetes: a pilot trial

**DOI:** 10.3402/fnr.v60.32594

**Published:** 2016-09-22

**Authors:** Gassan Darwiche, Peter Höglund, Bodil Roth, Ewa Larsson, Trygve Sjöberg, Björn Wohlfart, Stig Steen, Bodil Ohlsson

**Affiliations:** 1 Department of Clinical Sciences, Division of Internal Medicine, Lund University, Lund, Sweden; 2 Skåne University Hospital, Malmö, Sweden; 3 Department of Laboratory Medicine, Division of Clinical Chemistry & Pharmacology, Lund University, Lund, Sweden; 4 Skåne University Hospital, Lund, Sweden; 5 Department of Cardiothoracic Surgery, Clinical Sciences Lund, Lund University, Lund, Sweden

**Keywords:** anthropometry, metabolic control, interventional study, type 2 diabetes, Okinawan diet, Nordic diet, quality of life

## Abstract

**Background:**

Our hypothesis was that a modified diet would improve blood glucose control with beneficial impact on weight management and overall health in established diabetes.

**Objective:**

This prospective interventional study investigated the clinical effect of an Okinawan-based Nordic diet on anthropometry, metabolic control, and health-related quality of life (HRQoL) in Scandinavian type 2 diabetes patients.

**Design:**

Food was prepared and delivered to 30 type 2 diabetes patients. Clinical information along with data on HRQoL, blood samples, and urine samples were collected during 12 weeks of diet interventions, with follow-up 16 weeks after diet completion.

**Results:**

After 12 weeks of dietary intervention, a reduction in body weight (7%) (*p*<0.001), body mass index (*p*<0.001), and waist circumference (7.0 cm) (*p*<0.001) was seen. Improved levels of proinsulin (*p*=0.005), insulin (*p*=0.011), and fasting plasma glucose (*p*<0.001) were found already after 2 weeks; these improved levels remained after 12 weeks when lowered levels of C-peptide (*p*=0.015), triglycerides (*p*=0.009), total cholesterol (*p*=0.001), and low-density lipoprotein-cholesterol (*p*=0.041) were also observed. Insulin resistance homeostasis model assessment for insulin resistance was lowered throughout the study, with a 20% reduction in hemoglobin A1c levels (*p*<0.001) at week 12, despite reduced anti-diabetes treatment. Lowered systolic blood pressure (9.6 mmHg) (*p*<0.001), diastolic blood pressure (2.7 mmHg) (*p*<0.001), and heart and respiratory rates (*p*<0.001) were accompanied by decreased cortisol levels (*p*=0.015) and improvement in HRQoL. At follow-up, increased levels of high-density lipoprotein-cholesterol were found (*p*=0.003).

**Conclusion:**

This interventional study demonstrates a considerable improvement of anthropometric and metabolic parameters and HRQoL in Scandinavian type 2 diabetes patients when introducing a modified Okinawan-based Nordic diet, independently of exercise or other interventions. Through these dietary changes, anti-diabetes treatment could be decreased or cancelled.

The pandemic of type 2 diabetes mellitus and obesity, driven by an increasing worldwide adoption of energy-dense diets and sedentary lifestyles, is associated with significant comorbidities and healthcare costs and represents two of the biggest global health challenges of the modern world (1). Obesity plays a central role in occurrence of various diseases such as hyperinsulinemia, hyperglycemia, and dyslipidemia, and it has been strongly associated with insulin resistance in both normoglycemic subjects and individuals with type 2 diabetes mellitus (2). Insulin resistance exacerbates chronic hyperinsulinemia favoring anabolic metabolism that renders increased body weight, fosters carbohydrate cravings, and promotes insulin resistance, further promoting anabolic metabolism (3). Dietary intervention capable of lowering the insulin demand, improving insulin sensitivity, or both, is likely to reduce the incidence of disorders related to insulin resistance, as indicated by interventional studies (4, 5). These studies suggest weight reduction based on diet and exercise modifications to be important factors to reduce the risk of diabetes development. Whether dietary changes alone can play a crucial role in the prevention of diabetes is less clear, because it is difficult to determine the individual effectiveness of diet, independently of physical activity and weight loss.

The important features of healthy dietary patterns include a high intake of whole-grain and non-processed, plant-derived foods and a lower intake of red meat, meat products, sweets, salt, high-fat dairy, and refined grains and are shared by Mediterranean and Okinawan diets (6). These diets have been shown to improve metabolism, inflammation, and cardiovascular health in the population (7). Residents of Okinawa, the southernmost prefecture of Japan, are known for their long average life expectancy and high number of centenarians, with an accompanying low risk of age-associated diseases. Much of the longevity advantage in Okinawa is thought to be related to a healthy lifestyle. Part of this healthy lifestyle includes the traditional diet (8) that is low in calories yet nutritionally dense, particularly with regard to vitamins, minerals, and phytonutrients in the form of antioxidants and flavonoids (9).

Recently, our research group showed that an Okinawan-based single meal could promote a more favorable postprandial metabolic response and more pronounced satiety than a traditional meal in healthy subjects (10). Interventional studies investigating the effect of an Okinawan-based diet on Scandinavian type 2 diabetes patients has to our knowledge never been done previously. The aim of this study was to investigate the clinical effect of a modified Okinawan-based Nordic diet on anthropometry, metabolic control, and health-related quality of life (HRQoL) in Scandinavian type 2 diabetes subjects.

## Materials and methods

The subjects were treated according to the Helsinki declaration and the study was approved by the Regional Ethics Review Board at Lund University (2014/460). All subjects gave their written, informed consent before entering the study, and the study was monitored by an external monitor and registered at ClinicalTrials.gov data base (NCT02405806).

### Study population

**Fig. 1:**
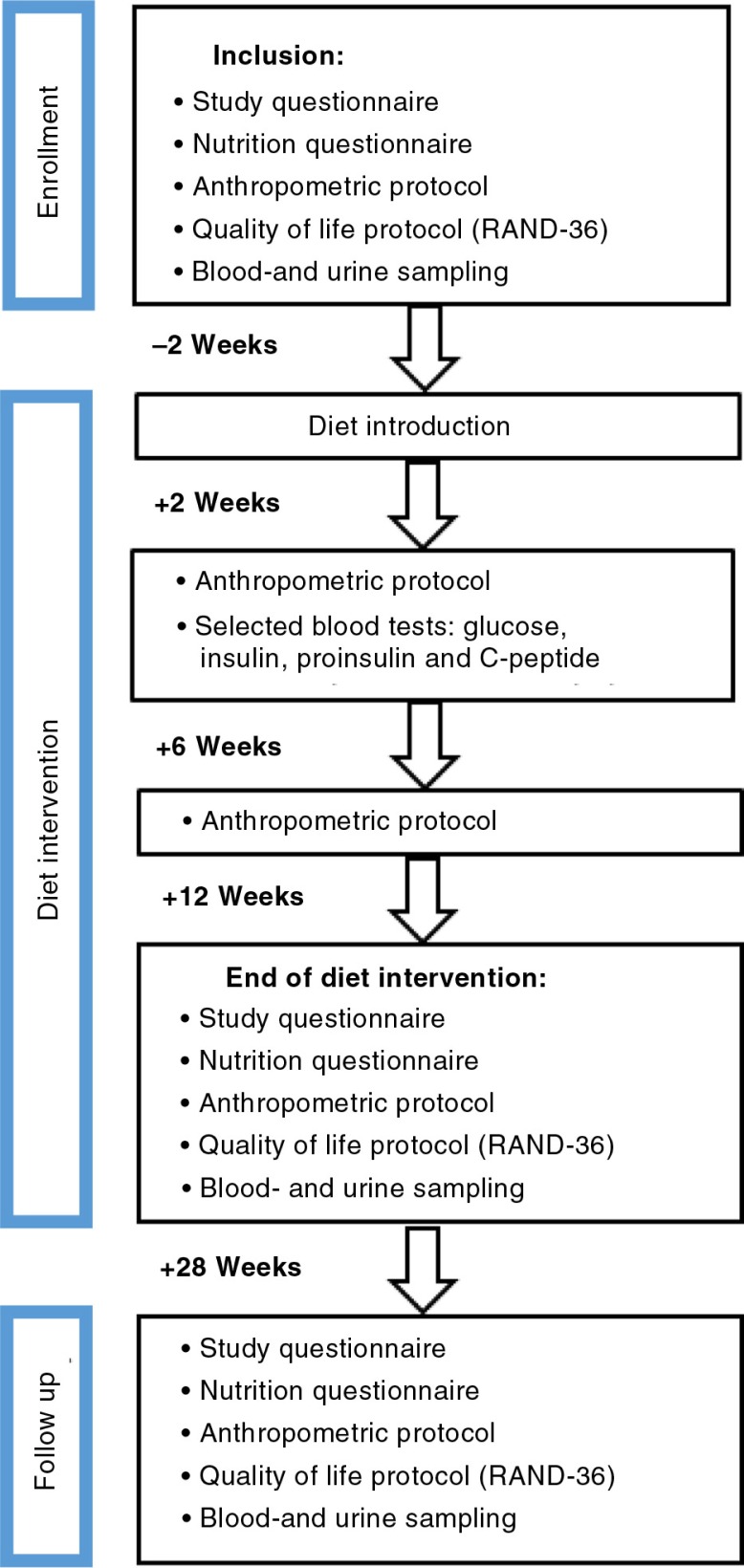
Flowchart of the design and phases of the Okinawan-based Nordic diet intervention study. Diet introduction followed a 2 week run-in period (2 weeks). The participants were followed up 16 weeks after the end of dietary intervention.

Thirty clinically diagnosed type 2 diabetes patients aged between 18 and 70 years were recruited at a primary health care centre in southern Sweden. Patients were to have both parents inborn in Scandinavia, to avoid possible influence of ethnicity on the study results. They were included in the study independently of body mass index (BMI) or anti-diabetes treatment regimen. Overall exclusion criteria were severe food allergy; type 1 diabetes; a prior major gastrointestinal surgery; and severe heart, pulmonary, cardiovascular, malignant, or psychiatric diseases. Patients with severe liver insufficiency, defined as spontaneous international normalized ratio (INR)>1.1, or severe renal insufficiency, defined as estimated glomerular filtration rate (eGFR)<30 mL/min/1.73 m^2^, were excluded (Fig. 1). The study started on February 2, 2015 and ended on September 18, 2015.

### Study design

The trial was designed as a clinical prospective study and was conducted during 12 weeks after introduction of an Okinawan-based Nordic diet, followed by another 16 weeks when participants were allowed to choose their own diet. Before entering the study, the patients were invited to an information meeting about the food composition and meal order. The subjects underwent a 2-week run-in period and were then followed by regular visits at 2, 6, 12, and 28 weeks after diet introduction, meeting the team of physicians and one nutritionist (Fig. 1).

A self-administered study questionnaire; nutrition questionnaire; and a HRQoL questionnaire, Research and Development 36-Item Health Survey (RAND-36) (11), along with information sheet and consent form, were sent home to the subjects and later collected and checked during their first visit. At the initial visit, the subjects underwent a clinical examination, and anthropometric data were collected along with blood and urine samples. All participants were instructed by a nutritionist (EL) on how to prepare their breakfast, based on the data from the nutrition questionnaire. Ready-made food for lunch, dinner, and snacks was delivered in a cooler bag to the subjects at home, three times a week and free of charge, along with written information and recipes for the final meal preparation. The participants were encouraged to maintain their regular physical activity habits throughout the intervention. No new dietary supplements such as fish oil, probiotics, or multivitamin drugs were allowed to be introduced during the study period. At most, one visit to a restaurant or change to another diet per week was allowed. Journeys or a stay for a long period at another place had to be discussed with the principal investigator (PI). Smoking habits were not changed during the study. Maximal intake of alcoholic beverages was set to 30 g ethanol/week. The nutritionist had a close contact with the subjects to support them and enhance compliance as much as possible.

At 2 weeks, anthropometric and clinical measurements were checked along with selected blood tests including fasting plasma glucose, C-peptide, insulin, and proinsulin, and a dietary follow-up was carried out with the ability to adjust the diet for energy content and composition when required. Anthropometric and clinical data were re-collected at half-time after 6 weeks. At completion of the dietary intervention at week 12 and at follow-up at week 28, a slightly shorter follow-up version of the study questionnaire, the nutrition questionnaire, the HRQoL questionnaire RAND-36 were administered, and anthropometric and clinical data and blood and urine samples were collected.

### Assessment of anthropometry and clinical variables

The investigation took place at Skåne University Hospital in Malmö. Assessments of standard physical and clinical variables were done under identical conditions by two clinically experienced physicians (BO and GD). Physical examination included cardiopulmonary, abdominal, and neurological examination as well as measurement of blood pressure, pulse, respiratory rate, weight, height, waist circumference, and assessment of BMI (kg/m^2^). Blood pressure was measured in the supine position after a 5-min rest with the arm at the level of the heart. Height was measured to the nearest centimeter, and weight to the nearest kilogram in subjects wearing light clothes, but not shoes, and BMI was calculated. Normal weight was defined as BMI<25 kg/m^2^, overweight as BMI ≥ 25 kg/m^2^ but <30 kg/m^2^, and obesity as BMI ≥ 30 kg/m^2^ (12). Waist circumference was measured to the nearest centimeter in a standing position after a gentle expiration, midway between the lower border of the rib cage and the superior border of the iliac crest (13). In addition, questions about intake of dietary supplements, vitamins, and probiotics; changes in medication; physical activity or routines; and any extraordinary events of daily life were completed.

### Diets

The meals and snacks were planned together with the kitchen of Igelösa Life Science AB and delivered to the subjects regularly free of charge, along with recipes and instructions on how to prepare the food. The breakfast was exempt; the subjects had to buy breakfast themselves. Three alternatives were suggested, depending on their ordinary breakfast ingested, described in the nutrition questionnaire. The diet was based on the traditional Okinawan diet (6, 14), but it was modified and developed in the kitchen to suit the Nordic diet with tastes and food components suitable for the Nordic population. The meal composition was close to a moderately low carbohydrate-rich diet, one of four recommended diets from the Swedish National Food Agency for patients with diabetes (15). These recommendations are also in line with international recommendations (16, 17). The diet consists of ordinary raws, but with minimal industrial processing. The food is based on traditional Nordic raws, for example, whole grains, vegetables, leguminous, root crops, fat fish, birds, fruits, berries, and nuts (Table 1). Simultaneously, the amount of sugar, white flour, read meat, processed meat, and dairy products is limited. Actually, to promote a low glycemic response, the content of high glycemic index (GI) foods was reduced radically (e.g. white flour and sugar) in favor of nutrients with low impact on the blood sugar (Table 1). The diet has a good nutritional supply including a mean calorie intake of about 1,900 kcal/day. The participants were allowed to eat three meals a day including breakfast, lunch, and dinner, and two snacks between meals consisting of a variety of fruits, berries, and seeds (Table 1). Organic food items were preferred whenever possible. At occurrence of cravings, the subjects were instructed to eat a third snack (e.g. carrots, boiled eggs, mackerel in tomato sauce, or cottage cheese with berries) to avoid eating fast carbohydrates. Gently chopped raw vegetables and green salad were to be ingested with the main meals; 100 g at breakfast, 150 g at lunch, and 150 g at dinner. The participants were instructed always to start with the vegetables and to eat slowly. Nutrition information is given in Table 2. Most participants had irregular meal intake, and many did not eat any breakfast, before entry in the study.

**Table 1:** Description of food items included in each food group in the Okinawan-based Nordic diet

Food group	
Root vegetables	Hot or cold. Red or yellow carrots as snacks or incorporated in the dish. Parsnips, parsley root, rutabaga, celery root, beetroot, artichoke, or sweet potatoes as part of the dishes.
Potatoes	Shredded in potatoes burger, fried raw.
Vegetables	Onion, leek, garlic, cabbage, cauliflower, broccoli, squash, eggplant, fennel, spinach, various types of salad, tomato, pepper, cucumber, mushroom, and asparagus.
Legumes	Fresh: green peas, sweet peas, and soybean (edamame).
	Dried: white and black beans, kidney beans, and lentils.
Nuts	Walnuts, almonds, and cashews.
Seeds	Sunflower seeds, pumpkin seeds, linseed, and sesame seed, as part of snacks served with fruits and berries.
Fruits and berries	Apple, orange, pear, strawberry, blueberry, lingonberry, dried apricots, and prunes. Coconut milk was used for cooking.
Meat products and poultry	Chicken, rooster, turkey, game meat (hart and deer), and ground beef.
Fish and seafood	Salmon, codfish, plaice, mackerel, herring, and prawns.
Egg	Eggs were used for cooking and as snacks.
Dairy products	Low-fat drinking milk (maximum 1.5% fat), filmjölk (a Swedish fermented product similar to yogurt) and low-fat yogurt (1.5% fat), hard cheese (17% fat), parmesan cheese, cottage cheese, and quark. Whipped cream (36% fat) and soy cream used for cooking, and Turkish yogurt (10% fat).
Fat and oil	Vegetable oil including rapeseed oil, sesame oil, and olive oil for cooking and dressing. Vegetable fat spread (60% fat) for bread.
Rice	Whole-grain rice and black Thai rice. A maximum of half a deciliter of uncooked rice per serving.
Cereals	Rolled oats, rye flakes, whole-grain whole-kernel rye, oat, barley, and wheat. Bread rich in whole grain, a maximum of two slices per day. Whole-grain pasta, bean paste, millet, oat bran, quinoa, and rye flour.
Beverages/liquids (non-alcoholic)	Recommended free amount of tap water or mineral water with the meals. Light products to be avoided. Tea, filtered coffee, or instant coffee.
Spices	Own preference with restrictions on salt.
Sweets and desserts	Homemade dark chocolate (>70%), bean truffles, coconut flakes, ginger, and prune cake. No refined sugar or sweetener was used, but instead natural sweet foods such as prunes, pears, and occasionally honey.
Alcoholic beverages	Maximal intake of alcoholic beverages was set to 30 g ethanol/week.

**Table 2:** Nutrition composition and daily mean intake of energy, nutrients, and food components of the modified Okinawan-based Nordic diet, compared with NNR 2012

Nutritional value	Unit	Calculated value	E%	Recommended NNR 2012
Total energy	kcal	1,866	–	–
Energy (excluding beverages)	kcal	1,629	–	–
Protein	g	95.0	23	10–20 E%
Fat	g	63.9	35	25–40 E%
Saturated fatty acids	g	18.7	10	<10 E%
Polyunsaturated fatty acids	g	14.9	8	5–10 E%
Mono-unsaturated fatty acids	g	17.8	10	10–20 E%
Carbohydrates	g	168.4	42	45–60 E%
Sucrose	g	23.5	6	<10 E%
Dietary fiber	g	35.9	4	25–35 g
α-Tocopherol	mg	1.9	–	–
β-Carotene	µg	9902.1	–	–
Retinol	µg	259.7	–	–
Vitamin A	µg	139.9	–	700
Vitamin D	µg	8.8	–	10
Vitamin E	mg	11.4	–	8
Thiamine	mg	1.1	–	1.1
Riboflavin	mg	1.2	–	1.2
Niacin equivalent	mg	34.5	–	14
Niacin	mg	19.7	–	14
Vitamin B6	mg	2.1	–	1.2
Folate	µg	386.1	–	300
Vitamin B12	µg	10.4	–	2
Vitamin C	mg	303.0	–	75
Sodium	mg	2401.1	–	2,300
Potassium	mg	3385.6	–	3,100
Phosphorus	mg	1446.7	–	600
Calcium	mg	840.4	–	800
Iron	mg	10.7	–	15
Magnesium	mg	317.4		280
Zinc	mg	9.7		7
Iodine	µg	34.9		150
Selenium	µg	61.0		50

NNR=Nordic nutrition recommendations.

The daily average calorie content of the meals is given in kilocalories, and the energy percentage (E%) is presented for protein, fat, saturated fatty acids, polyunsaturated fatty acids, mono-unsaturated fatty acids, carbohydrates, and sucrose. The results are calculated on the intake of a normal week. Recommendations for a traditional diet according to the NNR are shown for comparison (15). Recommendations for total daily energy intake are not given because they are individual and differ between subjects. Vitamins A and D and iodine may be underestimated due to lack of data.

### Study questionnaire

A validated study questionnaire, originally designed for the Malmö Preventive Project (18) and later slightly modified for the great population cohort of Malmö Diet and Cancer Study (19), was used for data collection. It contains questions about ordinary lifestyle factors and socioeconomic factors including information on origin, marital status, education, occupation, diet, and physical activity. In addition, a history of tobacco smoking, snuff habits, and alcohol consumption is included. The questions also cover a medical history of previous illness, disorders, or surgical operations; allergies; current medications; intake of dietary supplements and probiotics; menstrual and reproductive history; use of exogenous hormones for contraception and postmenopausal replacement therapy; and data on heredity factors.

### Nutrition questionnaire

A nutrition questionnaire was used at recruitment to collect information on ordinary food habits and energy intake. This basic information was used by the nutritionist to individually design the breakfast, together with the participants (Table 3). The questionnaire was repeated at the end of the intervention and at follow-up.

**Table 3:** Mean energy composition of the individual breakfast meals over the course of the study (*n*=30)

	Breakfast at baseline					Breakfast at week 12				
		
Subject no.	Energy (kcal)	Protein (g/E%)	Fat (g/E%)	Carbs (g/E%)	Fiber (g/E%)	Energy (kcal)	Protein (g/E%)	Fat (g/E%)	Carbs (g/E%)	Fiber (g/E%)
1	488	22/18	33/59	25/20	6/2	331	17/21	17/46	24/30	7/4
2	184	7/15	3/15	29/64	6/7	258	13/20	10/33	27/42	7/5
3	301	12/16	22/63	14/19	3/2	267	16/24	15/50	15/23	4/3
4	191	10/20	12/55	11/24	0.7/0.7	420	28/28	16/34	37/36	5/2
5	209	8/16	5/21	31/60	5/5	248	10/17	8/28	30/49	8/6
6	241	8/14	5/17	38/64	8/6	81	3/15	2/26	10/52	3/8
7	515	20/16	21/36	55/43	12/5	362	24/27	13/33	33/37	5/3
8	230	10/18	11/40	22/39	3/2	537	33/25	19/32	53/40	8/3
9	526	26/20	24/40	49/38	5/2	309	23/30	10/29	31/41	5/3
10	260	9/14	12/41	28/43	3/2	248	14/23	9/30	26/42	5/5
11	119	7/24	6/41	9/30	3/5	131	8/25	7/44	8/26	3/5
12	298	10/13	9/26	43/58	5/3	–	–	–	–	–
13	328	16/19	3/9	56/70	5/3	310	13/17	10/29	39/51	5/3
14	443	18/16	20/41	45/41	5/2	141	8/22	6/40	12/35	3/3
15	332	11/14	8/22	51/62	4/2	221	16/30	5/21	25/46	4/4
16	213	7/13	3/13	36/69	5/5	253	15/24	9/32	24/39	6/5
17	291	10/14	14/43	28/40	8/5	537	15/12	33/55	40/31	9/3
18	497	21/17	27/48	41/34	4/1	188	11/24	4/19	14/30	4/4
19	300	12/16	13/38	31/43	10/6	385	18/19	15/35	42/44	8/4
20	343	13/16	16/40	33/40	8/4	175	9/21	9/44	14/32	4/4
21	383	15/16	11/26	51/54	8/4	299	10/13	6/17	49/67	8/5
22	590	25/17	29/44	55/38	4/1	–	–	–	–	–
23	390	17/17	11/26	51/53	8.5/4	364	14/16	15/37	38/42	9/5
24	555	19/14	26/42	56/41	11/4	488	25/21	15/27	58/48	9/3
25	502	25/20	18/32	57/46	5/2	341	19/23	17/43	27/32	5/3
26	212	10/19	4/17	31/59	6/5	265	15/23	3/11	39/60	9/6
27	465	20/18	18/32	53/46	4/2	355	24/27	14/35	31/36	5/3
28	290	15/21	17/53	18/25	2/1	–	–	–	–	–
29	153	6/17	3/19	24/65	4/4	305	15/20	9/27	38/51	8/5
30	454	21/19	16/31	52/47	7/3	374	20/21	11/26	45/50	7/3

Two subjects (12 and 22) did not complete the study, and one subject (28) did not submit the logbook at the end of the study.

### Health-related quality of life questionnaire (RAND-36)

The validated self-report questionnaire: RAND 36-Item Health Survey was applied to evaluate quality of life (11). This instrument has been used to measure HRQoL in many various diseases, and normal references values are available (20). It comprises 36 items that assess eight health concepts: physical functioning, role limitations caused by general health perceptions, physical health problems, role limitations caused by emotional problems, social functioning, emotional well-being, energy/fatigue, and pain. The percentage scores range from 0% (lowest or worst possible level of functioning) to 100% (highest or best possible level of functioning). The few minor differences between 36-Item Short-Form Health Survey (SF-36) and RAND-36 have been described by Hays et al. (21).

### Blood samples

Venous blood samples were taken in the morning between 07:45 and 09:00 h after a 10-h fast. All samples consisted of whole blood drained into 6-mL serum separation tubes, plasma separation tubes, and BD Microtainer tubes with EDTA (BD Biosciences, Franklin Lakes, NJ). Blood chemistries were analyzed continually during the study. Remaining samples were stored at −80°C after immediate cooling and centrifugation of the blood at 3,000 rpm for 5 min, for later analyses.

Blood chemistries at baseline and at weeks 12 and 28 included blood count (hemoglobin, leukocytes, and thrombocytes), iron status (iron, total iron-binding capacity, and transferrin), C-reactive protein [CRP], kidney function tests (calcium (Ca), magnesium (Mg), potassium (K), sodium (Na), creatinine, and eGFR), liver function tests (alanine transaminase, aspartate aminotransferase, alkaline phosphatase, gamma glutamyl transpeptidase [GGT], bilirubin, albumin, and INR), cobalamin, and folate. Metabolic analyses measured were C-peptide, insulin, glucose, glycosylated hemoglobin A1c (HbA1c), and lipid profile (cholesterol, high-density lipoprotein [HDL], low-density lipoprotein [LDL], and triglyceride), and apolipoproteins A1 and B. Apolipoproteins A1 and B were used to calculate the apolipoprotein B/apolipoprotein A1 ratio as a risk predictor for cardiovascular events. Homeostasis model assessment for insulin resistance (HOMA2-IR) was calculated using the HOMA2 calculator version 2.2.3 (22, 23). As recommended by the HOMA2 calculator software, observations with extreme values of fasting plasma glucose (<3 or >25 mmol/L), serum C-peptide (<0.2 or >3.5 nmol/L), or both, were excluded. Serum insulin and C-peptide levels were determined using the one-step immune-complex sandwich assay with electrochemiluminescence immunoassay detection technique based on ruthenium derivatives (Elecsys^®^ insulin and C-peptide, Roche Diagnostics Ltd., Switzerland). Circulating stress markers were assessed by cortisol and thyroid-stimulating hormone (TSH). All samples were analyzed by standard methods at the Department of Clinical Chemistry, Skåne University Hospital, Malmö, Sweden.

At baseline and at week 2, fasting glucose, C-peptide, insulin, and proinsulin were measured. Proinsulin in serum was measured at the Department of Clinical Chemistry, Karolinska University Hospital, Stockholm, Sweden by an enzyme-linked immunosorbent assay (Proinsulin ELISA, Mercodia AB, Uppsala, Sweden) that uses a solid phase two-site enzyme immunoassay for photometric quantification of human proinsulin. There is no cross-reactivity with insulin or C-peptide.

### Urinary analysis for microalbuminuria

Urinary albumin and creatinine were measured using the Cobas Integra system (Roche Diagnostics Ltd., Rotkreuz, Switzerland). Urinary albumin was measured by the immunoturbidimetric method and urinary creatinine by the kinetic Jaffe reaction. The albumin/creatinine ratio was used to estimate microalbuminuria levels obtained from spot urine samples in the morning. Albuminuria was defined as the ratio of urine albumin to creatinine of ≥3.0 g/mol.

### Statistical analyses

A power analysis was performed based on previous studies of occasional subjects, and we found that at most nine subjects were needed to demonstrate clinically important differences in HbA1c, total cholesterol, systolic blood pressure, and weight and 18 subjects to demonstrate clinically important differences in diastolic blood pressure with 80% power at 5% significance level. We included 30 patients to take into account drop-outs. We tested hypotheses with linear mixed effect models to analyze continuous variables, with random intercept and unstructured covariance's for repeated measures within a patient, with visits as nominal fixed effect, using baseline as reference. We assumed that missing observations were unrelated to the observed value; that is, they were missing at random. In these analyses, predicted mean values and their 95% confidence limits are presented, together with estimates of changes from baseline and 95% confidence limits and *p* values for the changes. Descriptive statistics are presented as means and standard deviations for continuous variables and as counts or frequencies for categorical variables. Statistical calculations were done with MATLAB R2015a (The MathWorks Inc., Natick, MA). A *p* value <0.05 is considered statistically significant.

## Results

### Adherence to the diet and study protocol

The trial was initiated with 30 subjects. One of the subjects missed the 2-week follow-up, but otherwise completed the study. Two participants withdrew from the study at the half-time follow-up after 6 weeks: one because of inability to adapt to the new diet and the other because of work-related time constraints. Final samples were taken at withdrawal. Data obtained from non-completers and subjects with transitory infection (*n*=4), surgery (*n*=2), or depression (*n*=2) during the study period were not excluded. Baseline data collected at the time of the first clinical visit are given in Table 4.

**Table 4:** Patient characteristics at inclusion

Sex (female/male)	17/13	Antihypertensive medication (%)	63
Age (years)	57.5±8.2	Lipid-lowering medication (%)	47
BMI (kg/m^2^)	29.9±4.1	IBS (%)	13
Education (%)		Diabetes duration (years)	10.4±7.6
Primary school	16	HbA1c (mmol/mol)	61.6±17.6
High school	57	Diabetes management (%)	
College	27	Diet alone	7
Occupation (%)		Metformin	40
Employed	67	Sulfonylurea	3
Retired	17	Sulfonylurea and metformin	7
Sick leave	13	DPP-4 inhibitor and metformin	3
Unemployed	3	Metformin and insulin	27
Smokers (%)	23	Insulin	13
Snuff users (%)	23	Diabetes complication (%)	
Frequency of alcohol intake (%)		Retinopathy	27
None	10	Nephropathy	17
Once a month or less	50	Neuropathy	30
2–4 times a month	27	Gastroparesis	3
2–3 times a week	13	Macroangiopathy	17
Physical activity (%)			
Sedentary leisure time	7		
Moderate exercise during leisure	53		
Moderate regular exercise during leisure	27		
Regular exercise and training	13		

*n*=30. BMI=body mass index, IBS=irritable bowel syndrome, HbA1c=glycosylated hemoglobin, DPP-4=dipeptidyl peptidase-4. Age, BMI, total energy intake, diabetes duration, and HbA1c values are presented as means±standard deviation, and other values are presented as percentages.

The meals were well received by the majority of subjects. However, after 2 weeks, five subjects had to increase the caloric content of the food by an average of 146 kcal/day (distributed as 6 g of protein; 7 g of fat, of which 2 g was saturated fat; and 14 g of carbohydrates, including 2.5 g of dietary fiber) due to rapid weight loss. Higher energy requirements were identified related to physical activity at work and leisure time. All of these subjects went back at their own request to the initial calorie content of the meals after another 4 weeks at the half-time follow-up, as they felt sufficiently satiated. Four other subjects, all with irritable bowel syndrome, had to reduce the fiber content, whole-grain and root fruit content, or both, of the food because of troublesome bloating, abdominal pain, acid reflux, constipation, and in one case even diarrhea. In these cases, the protein content of the food was increased to maintain ordinary amount of calories, and at the half-time follow-up, the exaggerated symptoms were normalized. A reduction by half of the amount of whole grains entailed an increase in the protein proportion of 60 kcal/day.

Although encouraged to maintain their regular physical activity habits throughout the study period (Table 4), nine subjects had increased, and another four subjects had reduced, their regular physical activity by one level (e.g. from moderate exercise during leisure to moderate regular exercise during leisure or the opposite) at the end of the study.

### Anthropometric and clinical analyses

A progressive significant body weight reduction was seen at 2, 6, and 12 weeks after introduction of the new diet. The mean reduction of body weight and waist circumference at 12 weeks was 6.2 kg (*p*<0.001) and 7.0 cm (*p*<0.001), respectively. This weight loss was equivalent to 7% of total body weight at baseline. Mean BMI was reduced from 29.9 kg/m^2^ at baseline to 28.4 kg/m^2^ after 12 weeks (*p*<0.001), and 53% of the obese subjects (*n*=15) went from obesity to overweight, and 25% of the overweight subjects (*n*=12) went from overweight to normal weight. Despite some increase, the mean weight, BMI, and waist circumference remained significantly lower compared with baseline at 16 weeks after completion of dietary intervention (Table 5).

**Table 5:** Anthropometric analyses

Variable	Mean	Lower	Upper	Mean change	Lower	Upper	*p*
Weight (kg)							
Baseline	89.8	84.5	95.1	–	–	–	–
Week 2	86.7	81.4	92.1	−3.03	−3.61	−2.45	<0.001
Week 6	85.1	79.8	90.5	−4.66	−5.61	−3.72	<0.001
Week 12	83.6	78.1	89.0	−6.20	−7.61	−4.78	<0.001
Week 28	85.4	79.7	91.1	−4.40	−6.57	−2.24	<0.001
BMI (kg/m^2^)							
Baseline	29.9	28.4	31.3	–	–	–	–
Week 2	28.9	27.4	30.4	−1.00	−1.18	−0.81	<0.001
Week 6	28.3	26.8	29.8	−1.53	−1.83	−1.23	<0.001
Week 12	27.8	26.3	29.4	−2.05	−0.52	−1.57	<0.001
Week 28	28.4	26.8	30.0	−1.47	−2.13	−0.82	<0.001
Waist circumference (cm)							
Baseline	107.3	103.4	111.2	–	–	–	–
Week 2	104.1	99.6	108.6	−3.19	−5.53	−0.85	0.008
Week 6	102.6	98.6	106.6	−4.67	5.88	−3.47	<0.001
Week 12	100.3	96.1	104.4	−7.02	−8.62	−5.42	<0.001
Week 28	101.7	97.6	105.9	−5.54	−7.11	−3.96	<0.001
Heart rate (beats/min)							
Baseline	80.50	77.13	83.87	–	–	–	–
Week 2	72.48	68.33	76.63	−8.02	−11.41	−4.63	<0.001
Week 6	68.73	64.30	73.16	−11.77	−15.50	−8.05	<0.001
Week 12	67.27	62.77	71.77	−13.23	−17.04	−9.42	<0.001
Week 28	67.88	63.32	72.44	−12.62	−16.50	−8.74	<0.001
Systolic blood pressure (mmHg)							
Baseline	140.17	134.72	145.61				
Week 2	136.63	130.20	143.06	−3.54	−7.65	0.58	0.091
Week 6	132.96	126.58	139.34	−7.21	−11.25	−3.17	<0.001
Week 12	130.55	124.39	136.71	−9.62	−13.30	−5.93	<0.001
Week 28	139.74	133.84	145.65	−0.42	−3.65	2.81	0.796
Diastolic blood pressure (mmHg)							
Baseline	82.33	78.73	85.93	–	–	–	–
Week 2	77.78	73.79	81.77	−1.58	−1.54	−0.62	0.001
Week 6	76.29	72.49	80.08	−1.55	−2.43	−0.68	<0.001
Week 12	74.88	71.03	78.73	−2.70	−3.57	−1.84	<0.001
Week 28	78.74	74.76	82.72	−1.75	−2.78	−0.72	0.001
Respiratory rate (breaths/min)							
Baseline	17.30	16.34	18.26	–	–	–	–
Week 2	15.72	14.64	16.80	−1.58	−2.54	−0.62	0.001
Week 6	15.75	14.74	16.75	−1.55	−2.43	−0.68	<0.001
Week 12	14.60	13.60	15.59	−2.70	−3.57	−1.84	<0.001
Week 28	15.55	14.40	16.69	−1.75	−2.78	−0.72	0.001

BMI=body mass index.

*n*=30. The mean values, mean changes, and 95% confidence interval with upper and lower limits are presented for anthropometric parameters at inclusion (baseline); at 2, 6, and 12 weeks after introduction of diet intervention; and at 16 weeks after the end of diet intervention (week 28). Comparisons were made between each time point and baseline by using linear mixed effect models. A value of *p*<0.05 was considered statistically significant.

There were no changes in lipid-lowering or antihypertensive medication, except titration of enalapril from 10 to 15 mg in one case at the start of the study. A significant reduction in the systolic and diastolic blood pressure could be seen after 2, 6, and 12 weeks. The diastolic blood pressure remained lower compared with baseline after 28 weeks of follow-up (Table 5). A significant progressive mean reduction in the heart and respiratory rates could be seen already 2 weeks after the diet introduction, persisting during the following weeks of intervention and 16 weeks after completion of dietary intervention (Table 5).

### Metabolic biomarkers

During dietary intervention, there was a significant reduction in the mean fasting plasma glucose values already after 2 weeks (*p*<0.001), and these levels were maintained until the end of the intervention, when the mean level of HbA1c was reduced by 20% (*p*<0.001). At week 28, the mean level of HbA1c remained lower than at baseline (Table 6). There was a 43% reduction of proinsulin levels (*p*=0.005) and insulin levels (*p*=0.011) after 2 weeks and a significant reduction in C-peptide levels after 12 weeks (*p*=0.015). These reductions were followed by reduced insulin resistance after 2 weeks, which remained after 28 weeks (Table 6). None of the participants increased their anti-diabetes treatment during the study. However, in 15 cases, the anti-diabetes medication had to be reduced gradually during the study period, and in two cases, including one on insulin treatment, the medication was cancelled. These reductions persisted 16 weeks later. Out of the remaining 12 diabetes patients on insulin, three got their insulin therapy cancelled and another eight had their insulin doses reduced by 27.6±16.4% on average. During the dietary intervention period, there was also a significant reduction in cortisol levels (*p*=0.015), but this reduction did not remain at the 28-week follow-up (Table 6).

**Table 6:** Metabolic control and biomarkers for disease risk

Variable	Mean	Lower	Upper	Mean change	Lower	Upper	*p*
Fasting glucose (mmol/L)							
Baseline	9.71	8.54	10.87	–	–	–	–
Week 2	7.90	6.62	9.18	−1.81	−2.51	−1.11	<0.001
Week 12	7.91	6.55	9.27	−1.80	−2.63	−0.96	<0.001
Week 28	9.28	7.71	10.85	−0.42	−1.58	0.73	0.466
HbA1c (mmol/mol)							
Baseline	61.57	56.42	66.72	–	–	–	–
Week 12	49.20	44.02	54.38	−12.37	−16.40	−8.33	<0.001
Week 28	54.36	48.83	59.90	−7.20	−11.68	−2.72	0.002
Proinsulin (pmol/L)							
Baseline	23.84	16.70	30.98	–	–	–	–
Week 2	13.69	6.46	20.92	−10.15	−17.15	−3.15	0.005
Insulin (mIU/L)							
Baseline	15.53	12.72	18.35	–	–	–	–
Week 2	12.73	9.89	15.57	−2.81	−4.96	−0.66	0.011
Week 12	11.67	8.85	14.48	−3.87	−5.99	−1.75	<0.001
Week 28	12.78	9.32	16.24	−2.75	−5.67	0.16	0.064
C-peptide (nmol/L)							
Baseline	0.99	0.81	1.17	–	–	–	–
Week 2	0.92	0.73	1.12	−0.07	−0.17	0.04	0.208
Week 12	0.88	0.69	1.07	−0.11	−0.21	−0.02	0.015
Week 28	0.88	0.66	1.09	−0.11	−0.25	0.02	0.108
HOMA2-IR (U)							
Baseline	3.00	2.50	3.51	–	–	–	–
Week 2	2.53	1.96	3.09	−0.48	−0.85	−0.11	0.012
Week 12	2.37	1.82	2.91	−0.64	−0.98	−0.30	<0.001
Week 28	2.61	2.05	3.16	−0.40	−0.75	−0.05	0.025
Cortisol (nmol/L)							
Baseline	367	337	397	–	–	–	–
Week 12	325	290	360	−41.20	−74.07	−8.34	0.015
Week 28	348	315	382	−18.34	−49.73	13.06	0.249
Triglycerides (mmol/L)							
Baseline	1.79	1.41	2.16	–	–	–	–
Week 12	1.49	1.09	1.89	−0.30	−0.52	−0.08	0.009
Week 28	1.96	1.46	2.46	0.17	−0.20	0.54	0.367
Cholesterol (mmol/L)							
Baseline	4.65	4.36	4.95	–	–	–	–
Week 12	4.22	3.87	4.57	−0.44	−0.69	−0.18	0.001
Week 28	4.71	4.37	5.05	0.06	−0.18	0.30	0.636
HDL (mmol/L)							
Baseline	1.22	1.10	1.35	–	–	–	–
Week 12	1.19	1.05	1.32	−0.04	−0.10	0.03	0.267
Week 28	1.34	1.20	1.47	0.11	0.04	0.18	0.003
LDL (mmol/L)							
Baseline	2.92	2.62	3.22	–	–	–	–
Week 12	2.68	2.33	3.03	−0.24	−0.48	−0.01	0.041
Week 28	2.82	2.49	3.16	−0.10	−0.30	0.11	0.356
GGT (µkat/L)							
Baseline	0.80	0.53	1.07	–	–	–	–
Week 12	0.58	0.30	0.85	−0.23	−0.41	−0.04	0.016
Week 28	0.53	0.17	0.89	−0.27	−0.57	0.03	0.073

HbA1c=hemoglobin A1c, HOMA2-IR=homeostasis model assessment for insulin resistance, HDL=high-density lipoprotein; LDL=low-density lipoprotein, GGT=gamma glutamyl transpeptidase.

*n*=30 subjects. The mean values, mean changes, and 95% confidence interval with upper and lower limits are given for fasting blood values of glucose and HbA1c; fasting serum levels of proinsulin, insulin, and C-peptide; insulin resistance as measured by HOMA2-IR (one missing value); and fasting plasma values of cortisol, triglycerides, cholesterol, HDL, LDL and GGT. Values are shown at inclusion (baseline); 2, 6 and 12 weeks after diet intervention; and 16 weeks after the end of diet intervention (week 28). Comparisons were made between each time point and baseline by using linear mixed effect models. A value of *p*<0.05 is considered statistically significant.

Twelve weeks after the dietary introduction, a significant improvement was seen in the lipid profile for triglycerides (*p*=0.009), total cholesterol (*p*=0.001), and LDL-cholesterol (*p*=0.041) (Table 6). This improvement did not remain at the follow-up, 16 weeks afterward. In contrast, a significant increase was observed for HDL-cholesterol at this time (*p*=0.003) (Table 6). The mean reduction in GGT was 0.23 µkat/L (*p*=0.016) at the end of the intervention period. No significant changes in the albumin/creatinine ratio or the apolipoprotein B/apolipoprotein A1 ratio was seen during or after diet intervention. Blood count, iron status tests, CRP, kidney function tests, liver function tests (except for GGT), TSH, cobalamin, and folate were not affected during the study period (data not shown).

### Health-related quality of life

Compared with baseline, the mean scores for the RAND-36 scale improved and were significantly higher for physical functioning (*p*<0.001), general health (*p*<0.001), vitality (*p*=0.011), social functioning (*p*=0.001), and mental health (*p*=0.003) after 12 weeks. The largest increase was in the social functioning and physical functioning scales. The increase in the mean scores for the vitality scale peaked at 16 weeks after the end of diet intervention (*p*<0.001). Even though the other scale scores had declined at this point, the mean increase in physical functioning, general health, and social functioning were still significantly improved relative to baseline (Table 7).

**Table 7:** Evaluation of quality of life using RAND 36-Item Health Survey

Variable	Mean	Lower	Upper	Mean change	Lower	Upper	*p*
Physical functioning							
Baseline	75.7	69.3	82.1	–	–	–	–
Week 12	86.5	80.1	92.9	10.8	4.9	16.7	<0.001
Week 28	85.3	78.4	92.2	9.6	3.2	16.1	0.004
Physical role functioning							
Baseline	74.2	60.7	87.6	–	–	–	–
Week 12	78.3	64.9	91.8	4.2	−9.8	18.1	0.555
Week 28	73.3	57.7	88.8	−0.9	−16.9	15.1	0.911
Bodily pain							
Baseline	72.8	62.9	82.8	–	–	–	–
Week 12	79.7	69.1	90.3	6.9	−1.6	15.3	0.109
Week 28	80.3	69.1	91.5	7.5	−1.8	16.7	0.112
General health							
Baseline	58.7	51.2	66.1	–	–	–	–
Week 12	68.7	60.4	76.9	10.0	4.2	15.8	<0.001
Week 28	67.7	58.9	76.5	9.0	2.5	15.5	0.007
Vitality							
Baseline	56.2	48.1	64.3	–	–	–	–
Week 12	66.7	56.2	77.1	10.5	2.5	18.5	0.011
Week 28	68.4	59.3	77.5	12.2	6.1	18.4	<0.001
Social functioning							
Baseline	78.4	70.6	86.3	–	–	–	–
Week 12	89.3	79.8	98.8	10.9	4.3	17.4	0.001
Week 28	87.4	77.4	97.3	8.9	1.8	16.1	0.015
Emotional role functioning							
Baseline	81.1	71.3	90.9	–	–	–	–
Week 12	84.5	71.6	97.3	3.4	−9.1	15.8	0.592
Week 28	83.8	69.7	97.8	2.7	−11.0	16.3	0.699
Mental health							
Baseline	73.2	67.0	79.4	–	–	–	–
Week 12	82.0	75.3	88.7	8.8	3.2	14.4	0.003
Week 28	78.6	71.8	85.3	5.4	−0.4	11.1	0.067

*n*=30. The percentage scores range from 0% (lowest or worst possible level of functioning) to 100% (highest or best possible level of functioning). The mean values, mean changes, and 95% confidence interval with upper and lower limits are given for eight health concepts at inclusion (baseline); 12 weeks after diet intervention; and 16 weeks after the end of diet intervention (week 28). Comparisons were made between each time point and baseline by using linear mixed effect models. A value of *p*<0.05 is considered statistically significant.

## Discussion

This is the first study to investigate the effects of a modified Okinawan-based Nordic diet in Scandinavian type 2 diabetes subjects. In this 12-week intervention, the dietary intervention led to significant reductions in weight and waist circumference. Beneficial effects in metabolic parameters were achieved, including improvement in glucose-insulin homeostasis, insulin resistance, and lipid homeostasis during reduction of anti-diabetes treatment. The blood pressure was lowered and progressive reductions in the heart and respiratory rates were seen, along with reduction in cortisol levels and improvement of HRQoL.

Most data about food and nutrition are based on epidemiological studies or experimental studies using test solutions or substitution of one or two substances. Few studies are performed as interventional studies on complete meals. Consequently, there is a good understanding of individual food groups’ positive impacts on metabolic and cardiovascular risk factors, but there is no uniform general dietary strategy to prevent diabetes (24). Dietary interventions recommended by the European Society of Cardiology; the European Association for the Study of Diabetes, Diabetes and Nutrition Study Group (17); and the American Diabetes Association (16) emphasize an appropriate intake of total energy and a diet in which fruits, vegetables, whole-grain cereals, legumes, and low-fat protein sources predominate. Specific dietary recommendations include limiting saturated and trans fats and alcohol intake, monitoring carbohydrate consumption, and increasing dietary fiber and foods containing whole grains. The traditional dietary pattern in Okinawa encompasses high consumption of vegetables and legumes (mostly soy in origin); moderate consumption of fish products; low consumption of meat, meat products, and dairy products; and moderate alcohol consumption. The caloric intake is low, but the diet is rich in omega-3 fats, has a high monounsaturated-to-saturated fat ratio, and emphasis on low GI carbohydrates (14).

Low GI diets are associated with lower risk of type 2 diabetes and heart disease (25) and can be useful in the management of glucose levels in patients with diabetes (26). Whole-grain cereals and legumes have been reported to be generally associated with health-beneficial effects (27) including postprandial blood glucose control and reduction in the levels of circulating inflammatory biomarkers (28). Furthermore, epidemiological and clinical studies demonstrate that intake of dietary fiber and whole grains is inversely related to obesity (29), type 2 diabetes (30), cancer (31), and cardiovascular disease (32). Dietary fiber's beneficial influence on obesity has been attributed to a decreased caloric density of the foods, reducing the energy intake over time (29); decreased nutrient absorption (33); increased satiety (28); and a slower rate of food ingestion (34). The composition of indigestible carbohydrates has also been shown to diminish episodes of hyperglycemia and related metabolic risk variables through a mechanism involving colonic fermentation (28). Consequently, it has been suggested that low-GI foods, which in addition are rich in whole-grain constituents, could be particularly advantageous to diminish risk factors associated with insulin resistance (28).

Despite a mean calorie intake of 1,900 kcal/day in the Okinawan-based diet, the average weight reduction was 6.2 kg after 12 weeks, representing 7% of total body weight. Research shows that modest weight loss of 5–10% is associated with improved health outcomes, including lower blood glucose levels, lower blood pressure, and improved lipid levels (35). Observational studies have reported that weight loss more than 5 kg over a 10-year period reduced the risk of diabetes by 50% or more (36). One reason to the present described weight reduction may be an increased satiety found already after a single meal of the diet compared with a traditional meal (10). The Okinawan-based diet composition with more fiber and greater food volume demands more chewing, thereby constituting a slower eating pattern with increased mastication and slower gastric emptying (37), factors that all induce less feeling of hunger and desire to eat (38). Because of the lower carbohydrate content, energy composition is optimized by a higher proportion of protein. Protein is the most satiating nutrient, and high-protein intake directly activates the arcuate nucleus and nucleus tracts solitaries in the brain, resulting in a suppressed food intake and delayed gastric emptying (39). The concomitant reduction in waist circumference and GGT in this study indicate that weight loss was related to a decrease in abdominal obesity (40). This may be of importance, because a strong relationship between central obesity and cardiovascular disease has been found (41).

During the dietary intervention, the mean fasting plasma glucose values were significantly reduced along with reduced insulin resistance, and decreased HbA1c levels at the end of the intervention period, despite a considerable gradual reduction in anti-diabetes medication. Preproinsulin is processed to proinsulin, before conversion to insulin and C-peptide and stored in secretary granules, awaiting release on demand. If the demand for insulin triggered by insulin resistance reaches a certain threshold, an insufficiency of the cleavage capacity of β cells leads to an increased secretion of intact proinsulin in addition to the desired insulin molecule (42). A large cross-sectional study has confirmed that intact proinsulin is a highly specific marker for insulin resistance (43). Proinsulin and C-peptide have been demonstrated to be cardiovascular risk factors by stimulating plasminogen activator inhibitor-1 secretion to block fibrinolysis (44) and by pro-inflammatory and pro-atherogenic effects (42, 45). The markedly reduced levels of C-peptide, insulin, and proinsulin already 2 weeks after the dietary introduction suggest a rapid reduction of insulin resistance, as indicated by the results for HOMA2-IR. These immediate responses may rather depend on dietary changes than by weight changes, because only a single meal of the Okinawan diet attenuated postprandial levels of glucose, glucose-dependent insulinotropic polypeptide (GIP), C-peptide, and insulin in comparison with a traditional meal (10). Significant improvement was seen in the lipid profile for triglyceride, total cholesterol, and LDL-cholesterol in the present study, although the fat content was higher than recommended in traditional diabetes diet (16). This may depend on the improved glucose-insulin homeostasis and lower levels of GIP, because GIP together with hyperinsulinemia and hyperglycemia is strongly involved in lipid regulation in humans (46).

A significant reduction in cortisol levels could be observed, along with a progressive reduction in the heart and respiratory rates and lowering in systolic and diastolic blood pressure. Activation of the sympatho-adrenal medullary (SAM) system and the hypothalamo-pituitary-adrenal (HPA) axis increases cortisol levels, blood pressure and heart rate (47). Excessive activation of these pathways in response to physiological challenges, including food intake, has been linked to the development of numerous chronic diseases including cardiovascular disease, type 2 diabetes, anxiety, and depression (48). There are several potential mechanisms behind the observed phenomena of lowered stress levels in the present study. Animal studies suggest that obesity can be associated with increased HPA axis and SAM system in responses to stress (49), and human studies have shown that adiposity in men is associated with hyperactivity of the HPA axis after ingestion of a standardized meal consisting of 22% protein, 53% carbohydrates, and 25% fat (50). One study has shown that intake of meals with high carbohydrate content results in a significantly higher HPA axis response in women with predominant visceral body fat distribution, compared with women with peripheral body fat distribution and normal-weighted healthy controls (51). Both the weight reduction and the lower carbohydrate load in the Okinawan-based Nordic diet may have contributed to the reduced stress level. Furthermore, data indicate a strong relationship between obesity and HRQoL, and even a small weight reduction leads to significant improvements in HRQoL (52).

Although caloric restriction is an effective method of losing body weight and body fat, it becomes more challenging if compliance to the diet cannot be maintained. The impact of home delivery and the pre-portioned meals may have contributed to adherence to dietary restrictions during intervention. However, on the 16-week follow-up after diet intervention, the weight, BMI, waist circumference, insulin resistance, and HbA1c remained significantly lower than at baseline, even with sustained reduction in anti-diabetes treatment. Also, diastolic blood pressure and heart and respiratory rates remained lower than at baseline, and a significant increase in HDL-cholesterol was observed at this time. This delayed effect in the improvement of HDL-cholesterol is difficult to explain, but it could be attributed to a continued generally healthier dietary pattern, increased physical activity after the completion of the dietary intervention, or both. Actually, we assume these residual beneficial effects in part to be related to subject's adoption to changed eating habits (e.g. changing breakfast habits, diminished meal size, continuing eating fruit and vegetables, and regular meals). Participants were instructed always to start with the vegetables, to eat slowly and to drink water with the meals, to induce satiety and less intake of energy-rich food. Evidence to this advice comes from studies in which increasing the vegetable portion on the plate by substituting it for meat and grain significantly increased vegetable intake and reduced the energy intake (53, 54).

It remains to determine to what extent the beneficial role depends on dietary composition or weight reduction. Even though some of our findings support an influence of dietary changes, including an immediate metabolic response after diet introduction the first weeks in line with a single meal evaluation of Okinawan-based diet and traditional diet (10), we presume additional beneficial effects as a result of weight changes. A limitation of the present study was that no control group was included. It is a challenge to construct an appropriate control group in open, non-blinded studies. Instead, the participants were characterized at baseline and followed during and after diet intervention, thus being their own controls. A control group provides some evidence that changes occurring over time were not the result of natural temporal trends or of unmeasured events that occurred contemporaneously with the exposure under study. A similar diet interventional study, using a control group advised to follow their habitual diet and physical activity, did however not show any effects on these controls (55). Furthermore, these patients were already scheduled for regular, clinical follow-ups at their primary care center. Thus, the psychological benefit of being enrolled in a clinical study should be less than if patients without an established clinical contact had been included.

In conclusion, a modified Okinawan-based Nordic diet seems to be well accepted by the participants and the beneficial health effects is considerable in type 2 diabetes. The dietary intervention led to significant reductions in anthropometric and metabolic parameters with an improvement of HRQoL. Further studies have to be conducted to examine its potential to be applied for practical use in a larger Scandinavian population to reduce the risk for metabolic syndrome and associated diseases.
